# Human uterine leiomyoma-derived fibroblasts stimulate uterine leiomyoma cell proliferation and collagen type I production, and activate RTKs and TGF beta receptor signaling in coculture

**DOI:** 10.1186/1478-811X-8-10

**Published:** 2010-06-10

**Authors:** Alicia B Moore, Linda Yu, Carol D Swartz, Xaiolin Zheng, Lu Wang, Lysandra Castro, Grace E Kissling, David K Walmer, Stanley J Robboy, Darlene Dixon

**Affiliations:** 1 Cellular and Molecular Pathology Branch, National Toxicology Program, National Institute of Environmental Health Sciences (NIEHS), National Institutes of Health (NIH), Department of Health and Human Services (DHHS), Research Triangle Park (RTP), NC 27709, USA; 2 Integrated Laboratory Systems, Incorporated, RTP, NC 27709, USA; 3 Biostatistics Branch, NIEHS, NIH, DHHS, RTP, NC 27709, USA; 4 Department of Obstetrics and Gynecology, Duke University Medical Center (DUMC), Duke University, Durham, NC 27710, USA; 5 Department of Pathology, DUMC, Duke University, Durham, NC 27710, USA

## Abstract

**Background:**

Uterine leiomyomas (fibroids) are benign smooth muscle tumors that often contain an excessive extracellular matrix (ECM). In the present study, we investigated the interactions between human uterine leiomyoma (UtLM) cells and uterine leiomyoma-derived fibroblasts (FB), and their importance in cell growth and ECM protein production using a coculture system.

**Results:**

We found enhanced cell proliferation, and elevated levels of ECM collagen type I and insulin-like growth factor-binding protein-3 after coculturing. There was also increased secretion of vascular endothelial growth factor, epidermal growth factor, fibroblast growth factor-2, and platelet derived growth factor A and B in the media of UtLM cells cocultured with FB. Protein arrays revealed increased phosphorylated receptor tyrosine kinases (RTKs) of the above growth factor ligands, and immunoblots showed elevated levels of the RTK downstream effector, phospho-mitogen activated protein kinase 44/42 in cocultured UtLM cells. There was also increased secretion of transforming growth factor-beta 1 and 3, and immunoprecipitated transforming growth factor-beta receptor I from cocultured UtLM cells showed elevated phosphoserine expression. The downstream effectors phospho-small mothers against decapentaplegic -2 and -3 protein (SMAD) levels were also increased in cocultured UtLM cells. However, none of the above effects were seen in normal myometrial cells cocultured with FB. The soluble factors released by tumor-derived fibroblasts and/or UtLM cells, and activation of the growth factor receptors and their pathways stimulated the proliferation of UtLM cells and enhanced the production of ECM proteins.

**Conclusions:**

These data support the importance of interactions between fibroid tumor cells and ECM fibroblasts *in vivo*, and the role of growth factors, and ECM proteins in the pathogenesis of uterine fibroids.

## Background

Uterine leiomyomas (fibroids; myomas) are the most common benign tumors of the female reproductive tract [1] and can cause reproductive problems leading to hysterectomy. Although the exact cause of these tumors remains unknown, steroid hormones and growth factors and/or their receptors have been reported to play a pivotal role in their development [2].

Currently, there are few studies that have investigated the significance of the "inherent" unique composition of these tumors in the pathogenesis of uterine leiomyomas. These firm, circumscribed masses are known to possess a smooth muscle component, and may often have a significant extracellular matrix (ECM). The ECM of fibroids consists of fibroblasts, often termed myofibroblasts, and reportedly producing a predominance of collagen types I and III [3]. The "fibrous/collagenous" component that exists in these tumors, lends to the use of the colloquial derived terminology "fibroid". The ECM may provide a reservoir for growth factors, cytokines, chemokines, angiogenic and inflammatory response mediators, and proteases produced by tumor cells, that are known to regulate events such as cell growth and differentiation, and ECM turnover, which are critical to leiomyoma growth and regression [4-7]. Furthermore, it has been suggested that, in general, the growth of tumors is dependent on interactions between multiple inter-dependent cell types [8].

Over the years, advances have been made to gain a better understanding of cell-cell interactions for various cancers and other disease processes using a variety of models ranging from simple, such as co-culture and inbred/outbred rodent models, to the more sophisticated transgenic/knockout and xenograft models. It has been recognized that the interaction between tumors cells and the stromal compartment may play a significant role in cancer progression/proliferation. It is speculated that tumor development is caused by genetic alterations, in part, and tumor progression results from communication between neoplastic cells and their microenvironment [9].

Although the cellular, as well as, molecular mechanisms of tumor progression are unclear, it is believed that the tumor microenvironment can directly influence tumor development. The microenvironment includes fibroblasts, which represent the most abundant cell type in the tumor stroma [9] and plays an important role in cancer development and progression [10]. The microenvironment of neoplastic cells may provide signals that regulate transcription factors [10]. Fibroblasts may interact with neoplastic cells and produce ECM [9], and may induce tumor cells to produce/secrete a variety of soluble factors or proteins, such as growth factors, into the ECM. Furthermore, fibroblasts and the ECM in tumors may influence tumor progression [9].

Due to the abundance of ECM often observed in fibroids, we hypothesized that interactions between leiomyoma smooth muscle cells and fibroblasts of the ECM are important in the growth of these tumors and in the production of growth factors and ECM proteins. In this study, we used a two-chamber coculture system to mimic the *in vivo *condition occurring in uterine leiomyomas of women, to investigate whether human uterine leiomyoma-derived fibroblasts could stimulate cell proliferation of human uterine leiomyoma cells and the production of ECM proteins. We also evaluated if this enhanced uterine leiomyoma cell proliferation could be due to the induction of growth factors and activation of growth factor receptors and downstream effectors. Additionally, we determined if increased production of growth factors that are important in fibrogenesis and activation of their receptor signaling pathways could result in enhanced collagen type I synthesis, a major component of the ECM of leiomyomas *in vivo*. Through the development of an *in vitro model*, we can acquire a better understanding of the leiomyoma microenvironment, and determine the component(s) that play a pivotal role in the pathogenesis of uterine fibroids, as well as, better define the role of growth factors and ECM proteins in fibroid development.

## Results

### Intermediate Filaments and Cell Surface Markers

**Figure 1:**
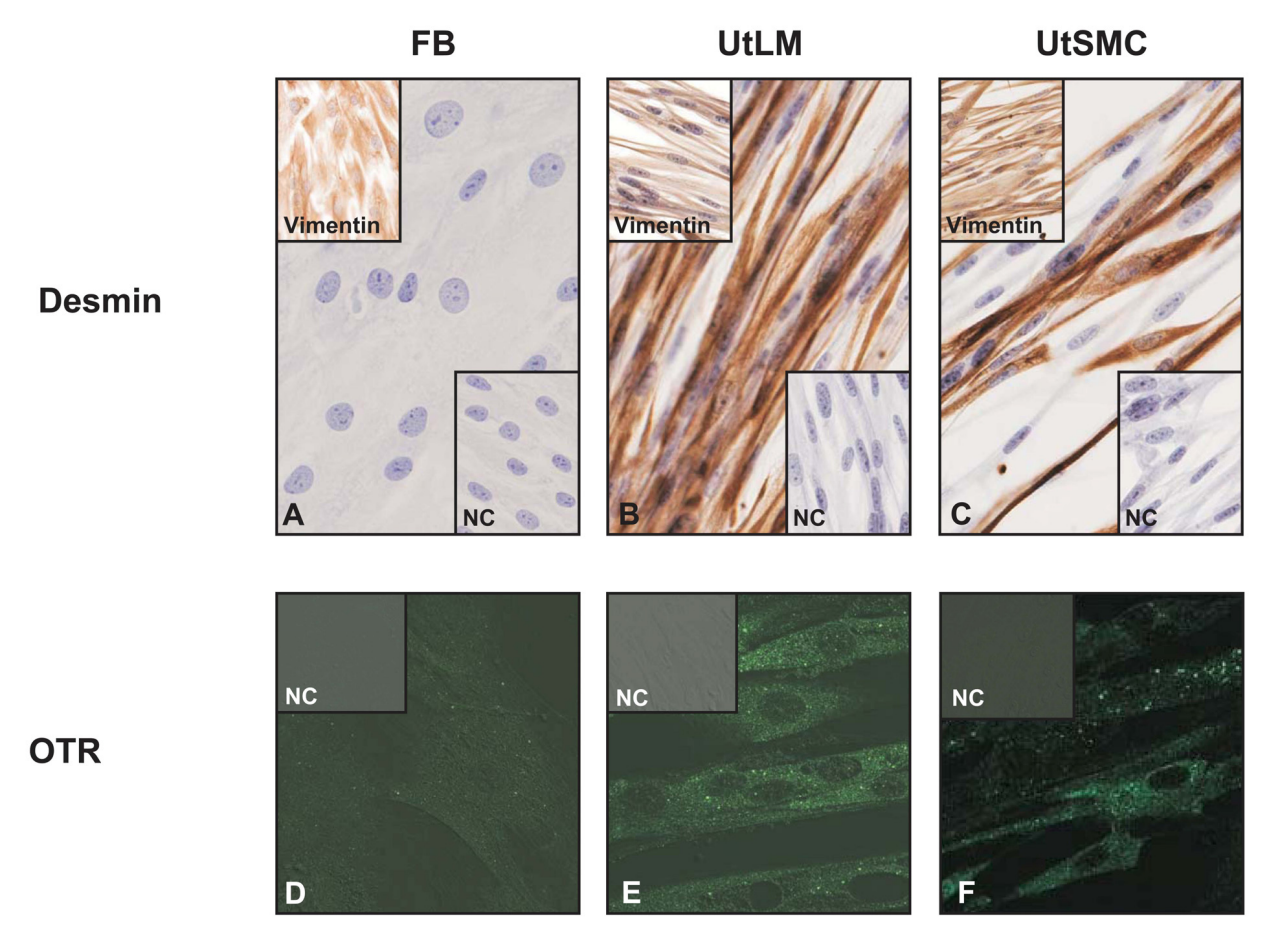
**Desmin and Vimentin**. (A) Uterine leiomyoma-derived fibroblasts (FB) stained negatively for desmin (center) and positively for vimentin (upper left). Inset lower right: Negative Control (NC) = Normal Mouse Serum. (B) UtLM cells and UtSMC (C) stained positively for desmin (center) and vimentin (upper left). Inset lower right: NC = Normal Mouse Serum. **OTR. **(D) Leiomyoma-derived FB was negative for the OTR. Inset upper left: NC = Normal Goat Serum. Positive staining of the OTR was observed in both UtLM cells (E) and UtSMC (F). Inset upper left: NC = Normal Goat Serum.

Human leiomyoma-derived fibroblasts (FB) were negative for desmin (Figure 1A, center), suggestive of a non-muscle origin and positive for vimentin (Figure 1A, upper left). The human uterine leiomyoma (UtLM) cells were positive for both desmin (Figure 1B, center) and vimentin (Figure 1B, upper left). Similarly, the human uterine smooth muscle (myometrial) cells (UtSMC) were positive for desmin (Figure 1C, center) and vimentin (Figure 1C, upper left). Normal mouse gamma globulin diluted in PBS (BioGenex) served as the negative control for both staining methods (Figures 1A-C, inset: lower right). The leiomyoma-derived fibroblasts (Figure 1D) were negative for the oxytocin receptor (OTR), indicating that the FB were not uterine smooth muscle cells. Both the UtLM cells (Figure 1E) and UtSMC (Figure 1F) were positive for the OTR, a G protein coupled receptor expressed in the myometrium [11] and leiomyomas [12,13]. Normal goat serum served as the negative control (NC) for the OTR staining method (Figures 1D-F, inset: upper left)

### Increased Cell Proliferation and Proliferating Cell Nuclear Antigen (PCNA) Labeling

**Figure 2:**
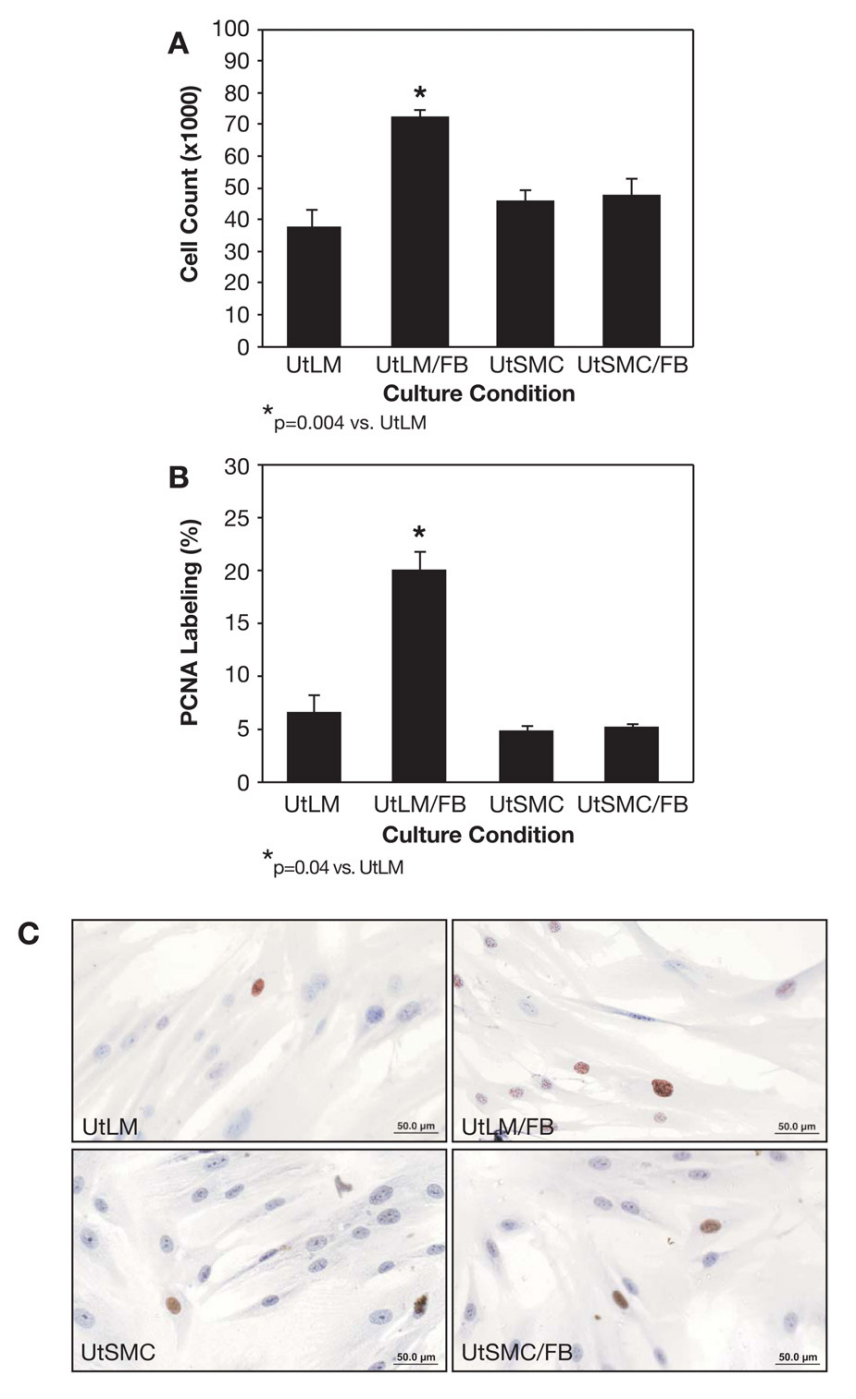
**Assessment of cell proliferation based on cell counts and PCNA labeling**. **(A) Cell proliferation**. There was a significant (p = 0.004) increase in the number of UtLM cells cocultured with fibroblasts (UtLM/FB) compared to UtLM cells cultured alone. There was no significant difference observed between UtSMC alone and UtSMC cocultured with FB **(B) PCNA labeling**. The percent PCNA labeling was significantly (p = 0.04) higher in UtLM/FB compared to UtLM cells cultured alone; however, there was no significant difference for UtSMC at both conditions. **(C) PCNA immunoexpression in UtLM cells and UtLM/FB**. PCNA was expressed in both cocultured and non-cocultured UtLM cells, as well as UtSMC, as indicated by brown nuclear staining. There was a significant increase in PCNA expression in the UtLM/FB compared to UtLM cells cultured alone, but not for UtSMC under similar conditions. All error bars represent SEM.

UtLM cells or UtSMC were cocultured with FB to determine if coculturing could stimulate proliferation of the respective uterine smooth muscle cell types (leiomyoma and myometrial cells). There was a significant (p = 0.004) increase in the number of UtLM cells when cocultured with FB compared to UtLM cells cultured alone (Figure 2A). However, there were no significant differences in the numbers of UtSMC cultured alone and UtSMC cocultured with FB (Figure 2A). There was a significant (p = 0.04) increase in the percent PCNA labeling of UtLM cells cocultured with FB (19.9%) versus UtLM cells cultured alone (6.45%) (Figure 2B). Also, increased immunoexpression of PCNA could be seen in UtLM cells cocultured with FB compared to UtLM cells alone (Figure 2C), No significant differences in percent PCNA labeling or PNCA immunoexpression were observed between the UtSMC cocultured with fibroblasts or UtSMC cultured alone (Figure 2B and 2C). These data support the concept that the increased PCNA labeling observed in cocultured UtLM cells in our study is dependent on the presence of leiomyoma-derived FB. Additional studies were done to determine the percent PCNA labeling in UtLM cells cocultured with other UtLM cells. There was no significant increase in the mean percent PCNA labeling of UtLM cells cocultured with UtLM cells (data not shown).

### Extracellular Matrix

**Figure 3:**
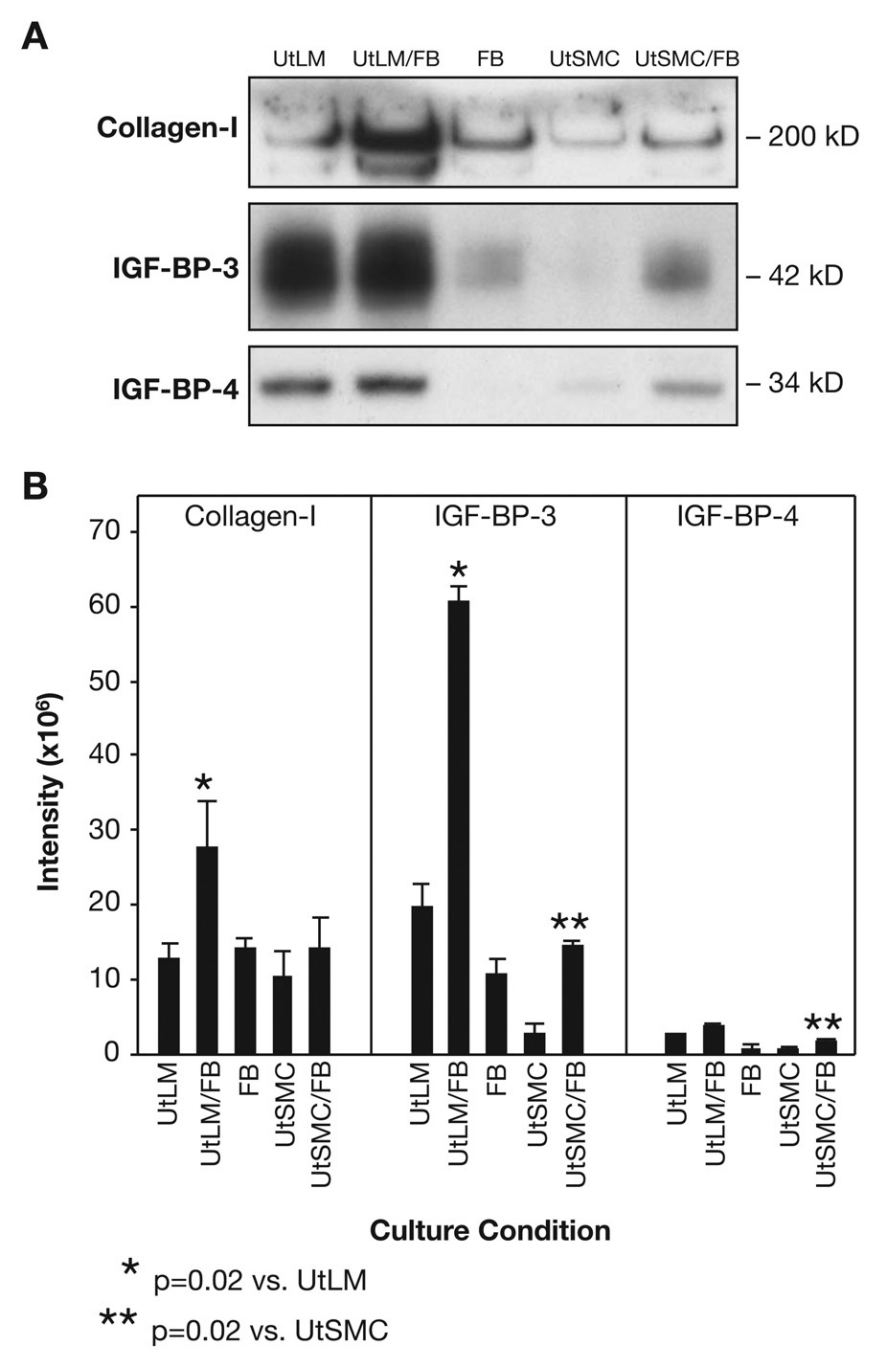
**ECM Proteins**. **(A) Western blots**. Collagen-I and IGF-BP-3 protein secretion in media of UtLM cells or UtSCM cultured in the presence (UtLM/FB or UtSMC/FB) or absence of FB. **(B) Densitometry**. Collagen I was significantly (p = 0.02) increased in the media of UtLM/FB cells compared to UtLM cells cultured alone. There was no difference between UtSMC versus UtSMC cocultured with FB. There was a significant (p = 0.02) increase in the expression of IGF-BP-3 in the cocultured UtLM cells compared to UtLM cells or FB cultured alone. Furthermore, IGF-BP-3 levels were also significantly higher in the cocultured UtSMC than UtSMC and FB cultured alone, but the level was minimal. There was a limited expression of IGF-BP-4 compared to IGF-BP-3 for both UtLM cells and UtSMC under cocultured or single cultured conditions. However, there was a significant (p = 0.02) increase in IGF-BP-4 expression in the cocultured UtSMC compared to UtSMC cultured alone. All error bars represent SEM.

There was an abundant secretion of collagen type I and IGF-BP-3 in the media of UtLM cells cocultured or cultured alone, compared to the media of UtSMC under cocultured or single cultured conditions, or FB cells cultured alone (Figure 3A). Collagen type I was significantly (p = 0.02) increased in the media of cocultured UtLM cells compared to UtLM cells cultured alone (Figure 3A and 3B). There was a significant (p = 0.02) increase in the secretion of IGF-BP-3 in the media of cocultured UtLM cells compared to UtLM cells cultured alone. The IGF-BP-3 levels were also significantly higher in the cocultured UtSMC than UtSMC cultured alone, although minimal. There was limited expression of IGF-BP-4 compared to IGF-BP-3 for both UtLM cells and UtSMC under cocultured or single cultured conditions; although, there was a significant (p = 0.02) increase in the expression of IGF-BP-4 in the cocultured UtSMC compared to UtSMC cultured alone (Figure 3A and 3B). An increase in IGF-BP-3 and -4 secretion was not detected in the media of UtLM cells cocultured with UtLM cells when compared to UtLM cultured alone or UtLM cells cocultured with fibroblasts (data not shown)

### Increased Secretion of GFs

**Figure 4:**
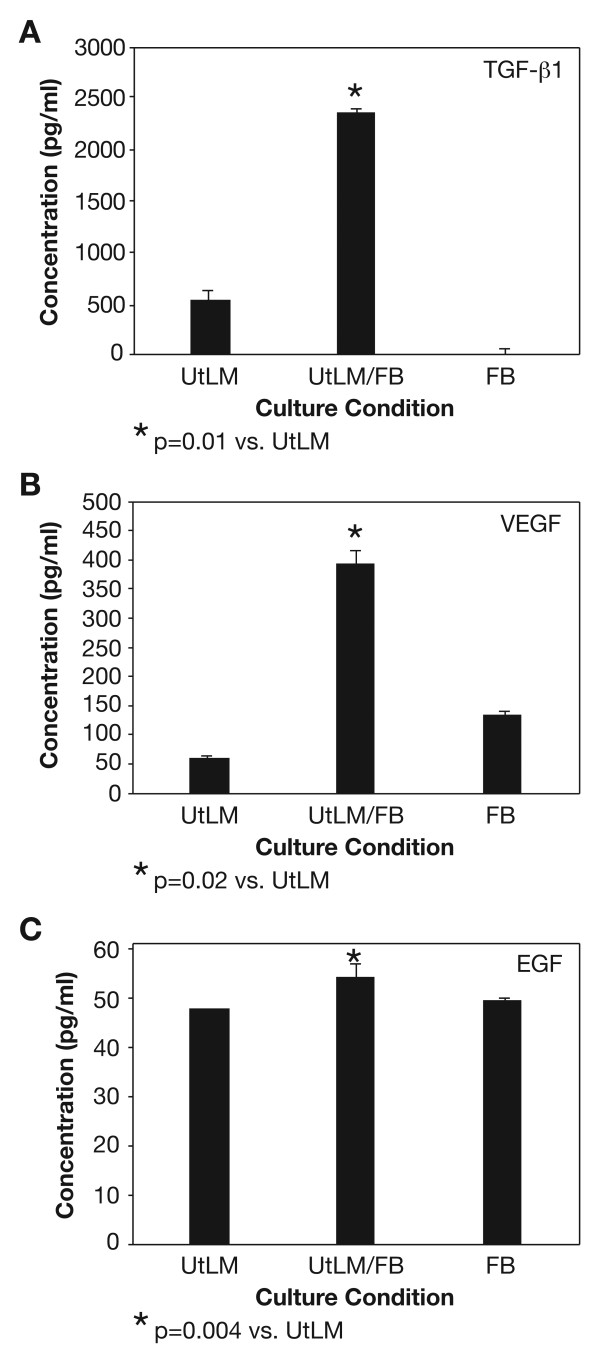
**Secretion of TGF-β1, VEGF and EGF in the media**. There was a significant increase in the concentration of (A) TGF-β1 (p = 0.01), (B) VEGF (p = 0.02) and (C) EGF (p = 0.004) in the media of UtLM/FB compared to UtLM cells or fibroblasts (FB) cultured alone by ELISA. All error bars represent SEM.

**Figure 5:**
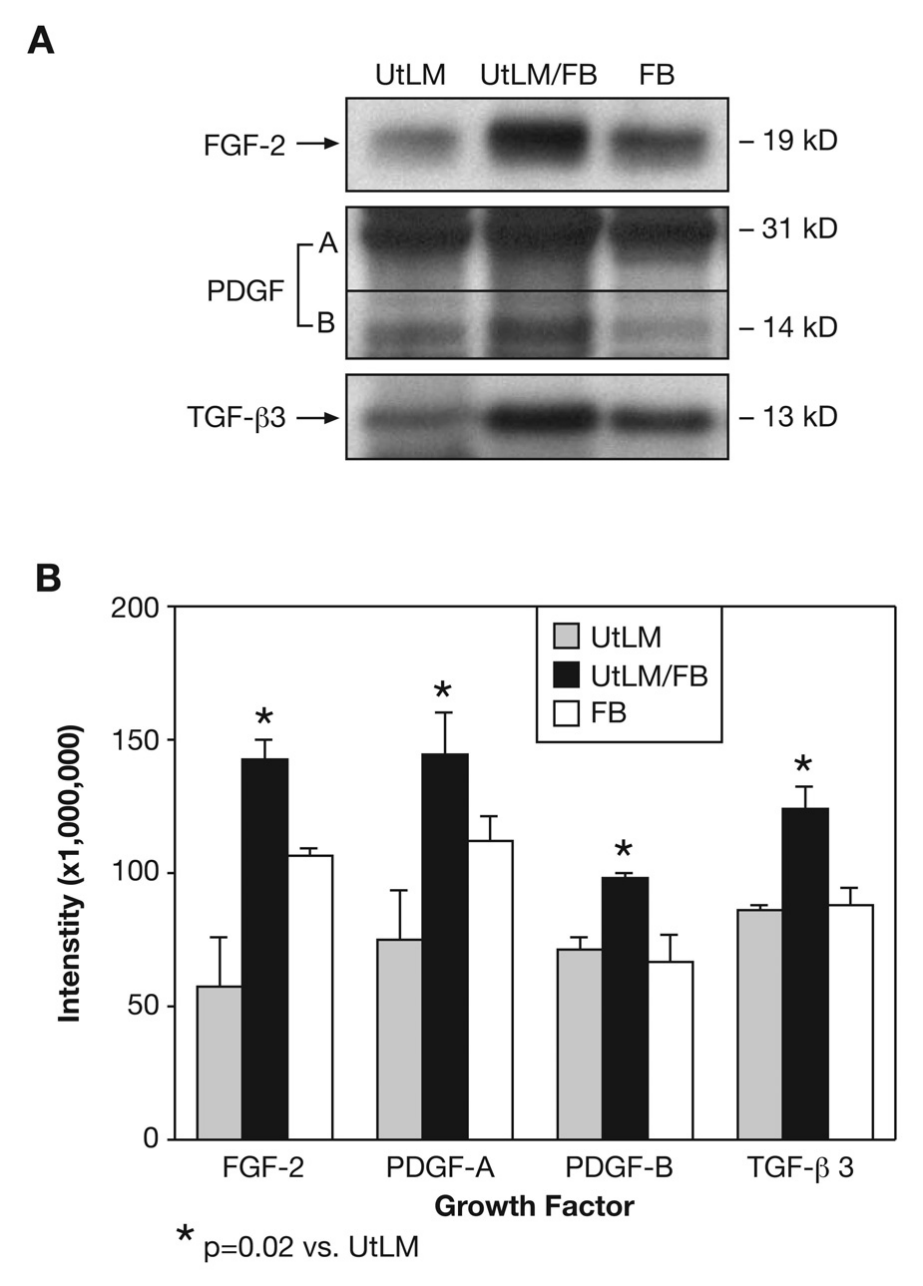
**Secretion of FGF-2, PDGF-A and -B, and TGF-β3 in the media**. **(A) Western blots of growth factor proteins. (B) Densitometric intensity of growth factor proteins**. There was a statistically significant (p = 0.02) increase in the secretion of FGF-2, PDGF-A and -B and TGF-β3 in the media of UtLM cells cocultured with fibroblasts (UtLM/FB) versus UtLM cells or FB cultured alone. All error bars represent SEM.

Because of the increased proliferation of cocultured UtLM cells and the induction of ECM proteins, which was not observed in cocultured UtSMC, we were interested in mechanisms, primarily cell signaling events that could possibly explain the differences in growth and ECM production between the cocultured UtLM cells, and the UtLM cells cultured alone. We investigated the secretion of growth factors in the media and found significantly increased levels of TGF-β1 (p = 0.01), VEGF (p = 0.02) and EGF (p = 0.004) in the media of cocultured UtLM cells compared to FB and UtLM cells cultured alone as determined by ELISAs (Figure 4A-C). Media levels of FGF-2, PDGF-A and -B, and TGF-β3 were also found to be significantly increased (p = 0.02) in cocultured UtLM cells compared to FB or UtLM cells cultured alone (Figure 5A and 5B) by western blot analysis; however, levels of insulin-like growth factor-I (IGF-I) were not detectable by ELISA or western blot analysis. When UtLM cells were cocultured with UtLM cells, increased secretions of the above GFs in media were not detected (data not shown)

### Increased Expression of pRTK

**Figure 6:**
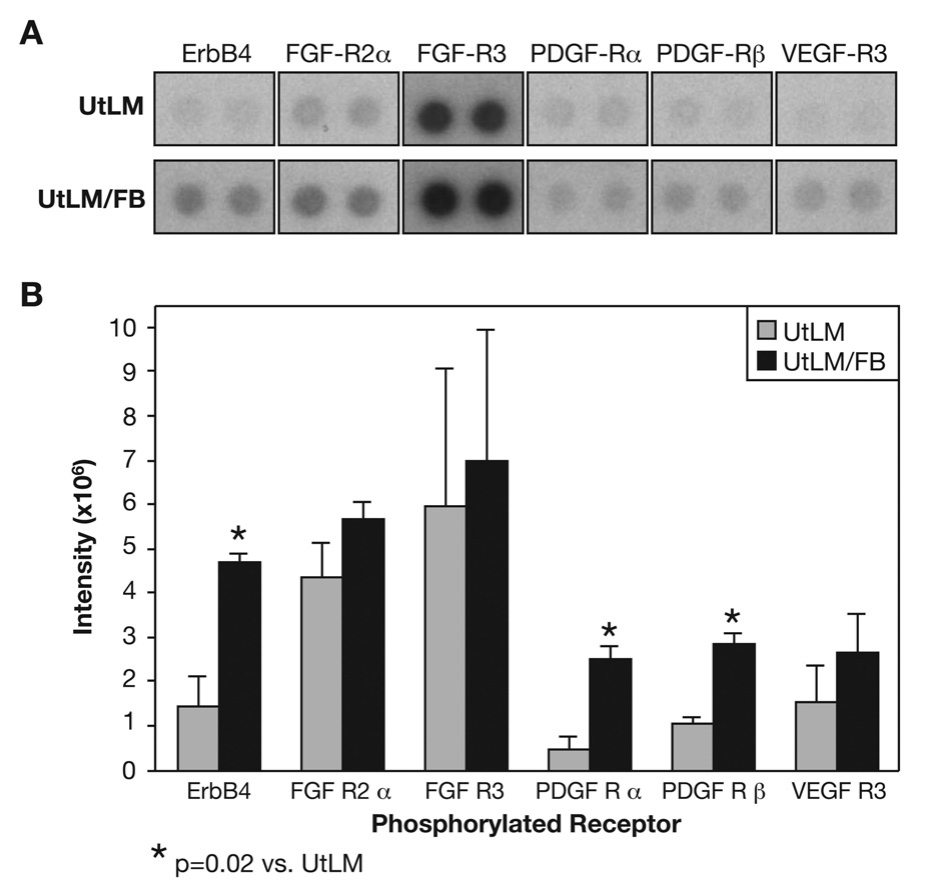
**Expression of phosphorylated (p)RTKs**. **(A) pRTK array**. The array detected activated RTKs for GFs under both culturing conditions **(B) Densitometry**. There was an overall increase in the expression of 5 pRTKs in the UtLM/FB compared to UtLM cells cultured alone. Significant (p = 0.02) increases in the phosphorylated receptors, ErbB4 and PDGFα/β, were found in the UtLM/FB compared to UtLM cells cultured alone. All error bars represent SEM.

Due to increased secretions of GFs observed in the media of cocultured UtLM cells, we were interested in assessing the activation of their respective receptors and signaling pathways. Using a pRTK array kit we found increased phosphorylation of 16 RTKs in cocultured UtLM cells compared to UtLM cells cultured alone. These receptor proteins belonged to EGF-receptor, FGF-receptor (FGF-R), hepatic growth factor-receptor, PDGF-receptor (PDGF-R), Tie, VEGF-receptor and Eph-R gene families, which all play important roles in proliferation, fibrogenesis, and angiogenesis during tumor development. For this study, we show the findings for 5 RTKs reported to be important in the growth/proliferation and/or ECM production in uterine leiomyomas. The other elevated RTKs determined in these assays are involved in primarily angiogenesis or other biological functions that will be addressed in future studies. There was increased expression of phosphorylated receptors v-erb-a erythroblastic leukemia viral oncogene homolog 4 (or ErbB4), FGF-R2α, FGF-R3, PDGF-Rα and β and VEGF-R3 in cocultured UtLM cells compared to UtLM cells cultured alone (Figure 6A and 6B); however, there was variability among the intensity values, resulting in a statistically significant (p = 0.02) increase in only the phosphorylated ErbB4, PDGF-Rα, and PDGF-Rβ (Figure 5B). Interestingly, the increased expression of these RTKs in the cell lysates was very similar to what we have observed in leiomyoma tissue [14].

### Increased expression of the downstream effector of RTK signaling, phosphorylated (p)-MAPK44/42

**Figure 7:**
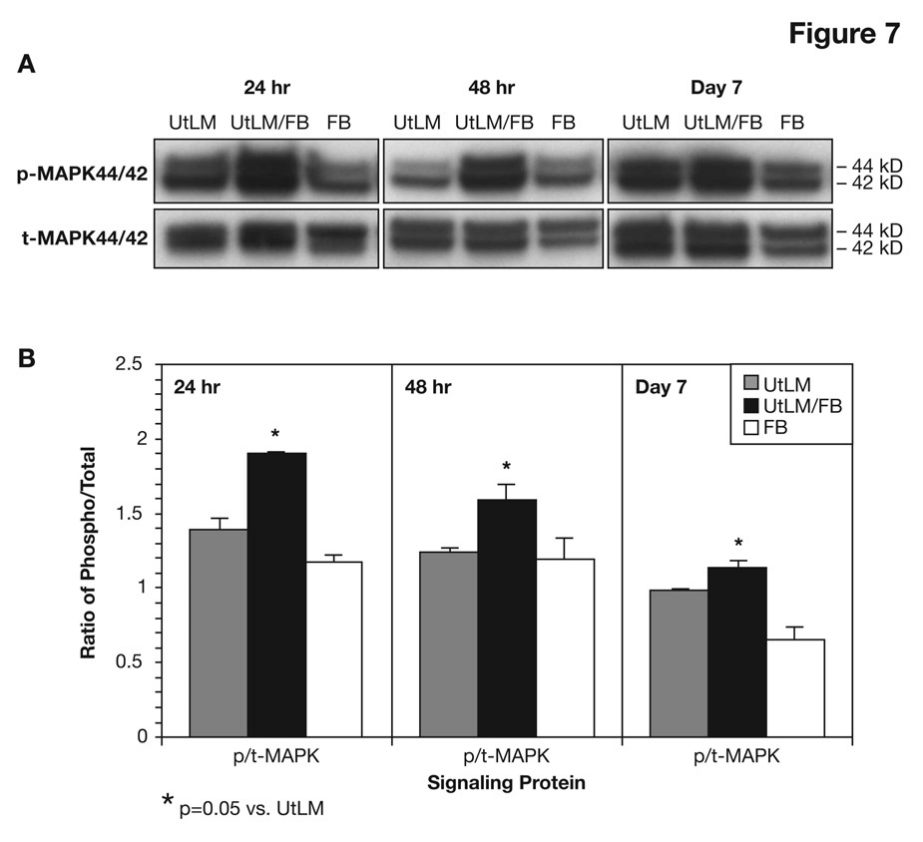
**Detection of phosphorylated (p)-MAPK44/42**. **(A) Western blots**. There was a significant increase in the expression of p-MAPK44/42 in the UtLM/FB compared to cultures of UtLM cells or FB alone at 24 h, 48h and day 7. **(B) Densitometry**. There was a significant increase in the expression of p-MAPK44/42 in cocultured UtLM cells compared to non-cocultured UtLM cells and FB at 24 h (p < 0.0001), 48 h (p = 0.05) and day 7 (p = 0.05). There were no differences observed between the culturing conditions for total (t)-MAPK44/42 expression. All error bars represent SEM.

There was a significant increase in expression of p-MAPK44/42 in cocultured UtLM cells compared to non-cocultured cells at 24 h (p < 0.05), 48 h (p < 0.05) and day 7 (p < 0.05) (Figure 7A and 7B), supporting the concept that increased secretion of GFs and activation of pRTKs observed in these cells results in enhanced downstream effector MAPK44/42 activation. The ratio of phosphorylated to total (p/t)-MAPK44/42 protein in cocultured and non-cocultured UtLM cells further indicates the increased expression level of p-MAPK 44/42 in the cells under cocultured conditions is due to the elevated phosphorylation of the protein not because of increased cell number (Figure 7B).

### Activation of the TGF-β receptor signaling pathway and Smad-2 and -3 proteins

**Figure 8:**
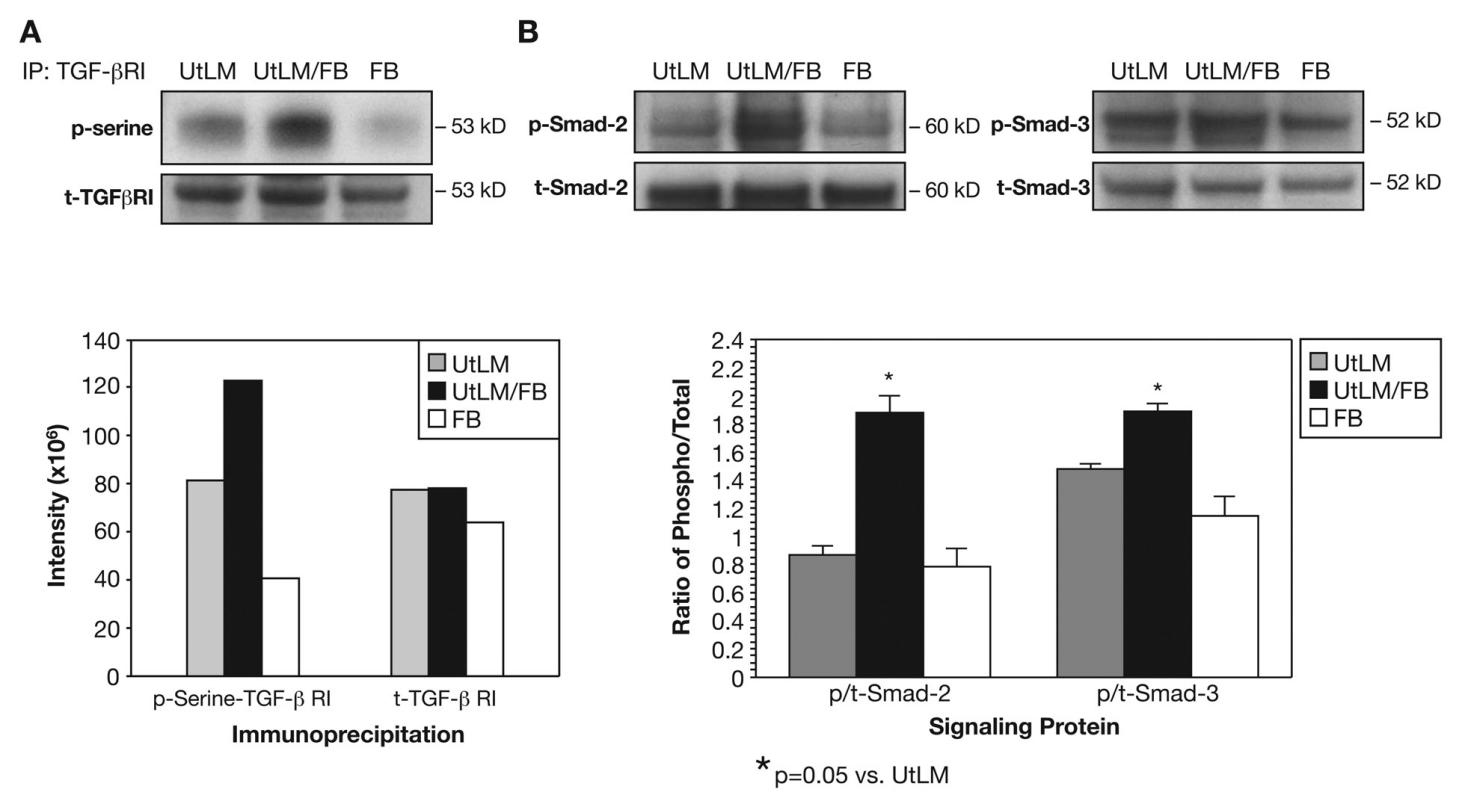
**Detection of activated TGF-βRI and downstream proteins, p-Smad-2 and -3 at 48 h**. **(A) Immunoprecipitation of TGF-**β**RI, blotted with anti-phospho-serine**. There was increased expression of phospho-serine in immunoprecipitated TGF-βRI in cocultures of UtLM/FB compared to cultures of FB or UtLM cells alone. There were no significant differences in total (t)-TGF-βRI expression between the culturing conditions. **(b) Western blots of p-Smad-2 and p-Smad-3 and t-Smad-2 and t-Smad-3**. Densitometric analyses showed a significant increase in the expression of phospho-Smad-2 (p = 0.001) and phosho-Smad-3 (p = 0.05) in UtLM/FB compared to UtLM cells or FB cultured alone. There was no significant difference in t-Smad-2 expression between UtLM/FB and UtLM cells cultured alone. All error bars represent SEM.

Due to the proliferation and excessive protein levels of collagen type I observed in cocultured UtLM cells, the TGF-β receptor signaling pathway and down stream effectors Smad-2 and -3 were investigated. We found that when TGF-βRI was immunoprecipitated from cocultured UtLM cells there was increased phosphorylation of serine sites on these cells compared to UtLM cells or FB cultured alone (Figure 8A). The ratios of p/t for the downstream effector proteins, phosphorylated Smad-2 and-3, were also significantly (p = 0.05) increased in cocultured UtLM cells compared to non-cocultured cells (Figure 7B). Total TGF-βRI, as well as, t-Smad-2 and-3, did not differ significantly between cocultured UtLM versus UtLM cells cultured alone (Figure 8B).

## Discussion

Although the exact etiology of uterine leiomyomas remains unknown, there is substantial evidence that hormones, such as estrogen and progesterone, partially regulate the growth of these tumors [15]. In addition, the growth-promoting effects observed in uterine leiomyomas may also be mediated by secondary effectors, such as growth factors, produced by uterine smooth muscle cells and fibroblasts [7,16]. Uterine leiomyomas often have a prominent fibrous component comprised of fibroblasts and various ECM proteins and enzymes that may also be important in promoting tumor growth [17,18]. Furthermore, evidence exists showing that interactions may occur between fibroblasts and tumor cells in promoting tumor growth [19-21].

In recent years, much effort has been made to improve the understanding of cellular processes involved in cell-cell interactions in various tumor cells, such as mammary carcinoma cells, using various models such as coculture, or conventional or genetically altered animals [20]. To date, there is an absence of studies that have investigated cell-cell interactions occurring in the microenvironment of leiomyomas. In this paper, we conducted experimental studies using a coculture model to gain a better understanding of the interactions between two cell types, leiomyoma cells and extracellular matrix fibrobasts, which are the major components of fibroids. We also show the importance of the interactions as it relates to growth factor, cell signaling, proliferation, and extracellular matrix production. To our knowledge, this is the first study whereby interactions of human UtLM cells and uterine leiomyoma-derived fibroblasts have been described.

Our findings show that uterine-leiomyoma-derived FB can stimulate the proliferation of UtLM cells, and production of collagen type I and IGF-BP-3 in the medium of UtLM cells cocultured with FB. This effect was not seen when UtLM cells were cultured alone or co-cultured with other UtLM cells, which indicates the microenvironment created by coculturing UtLM cells with tumor derived FB simulates the *in vivo *condition, and provides an experimental model that can be used to decipher the mechanisms of fibroids growth.

Recently, the tumor microenvironment has been recognized as the place of crosstalk between different cell types and cellular signaling [10,22]. Tumor cells are known to secrete a variety of proteins into the ECM microenvironment. In *in vitro *studies, many have found that growth factors individually or in combination stimulate uterine leiomyoma cell proliferation [15,16,23-25]. The proliferative or fibrogenic growth factors, such as VEGF, FGF-2/FGF-β, PDGF-A and -B, TGF-β1 and TGF-β3 have been previously reported to not only stimulate uterine leiomyoma cell proliferation in culture, but some have been reported to enhance collagen production [25]. It is believed that indeed, the pathogenesis of leiomyomas at the cellular and molecular levels involves the interactions of cytokines/growth factors, as well as ECM components, that all play a pivotal role in the development of these tumors [26]. Furthermore, it has been suggested that some of the sulphated glycosaminoglycans (GAGs), such as heparan sulfate and heparin in the ECM, are involved in creating a storage depot for biologically active molecules, such as growth factors and enzymes, and under conditions of hydrolytic degradation of the GAGs there may be a release of cytokines and other factors that may promote tumor cell growth and stimulate collagen biosynthesis [27]. In this study, we found that many of the growth factors associated with proliferation and fibrogenesis, such as EGF, TGF-β1, TGF-β3, VEGF, FGF-2, and PDGF-A and -B were increased in the media of cocultured UtLM cells, which suggested that these soluble factors are capable of stimulating the proliferation of UtLM cells and collagen I production. Our results are also consistent with other findings in that numerous growth factors and/or their receptors have been identified in myometrial and/or uterine leiomyoma tissues, and reported to be elevated in leiomyomas versus myometrium [4,16,23,25,28,29].

Microarray analyses have been done to characterize the gene expression profiles of leiomyoma and matched myometrial smooth muscle cells [30]. The collagen gene has been identified in leiomyoma smooth muscle cells and matched myometrial smooth muscle cells [30,31]. It has been demonstrated that the expression of collagen in these cells is regulated by TGF-β through the activation of MAPK [31]. Activation of receptor tyrosine kinases and TGF-β receptor, and phosphorylation of their downstream effectors, MAPK44/42 and Smad2/3, respectively, in this study indicates that increased levels of growth factors in cocultured media most likely initiated these signaling cascades. Although there are few reports on receptor tyrosine kinase signaling in uterine leiomyoma cells [14,32,33]), one study has shown that treatment of leiomyoma cells with 17-β estradiol results in enhanced secretion of PDGF, leading to the activation of the MAPK pathway and cell proliferation [32]. Expression of PDGF-A and -B was shown to be higher in leiomyoma tissue compared to normal myometrial tissue [34]. A study using reverse-transcriptase polymerase chain reaction (RT-PCR), recently found that the new PDGF subtype "C" mRNA was significantly upregulated in leiomyomas by 2.4-fold compared to adjacent normal myometrium, which was possibly due to involvement of abnormal collagen formation and angiogenesis that may have stimulated uterine leiomyoma growth [35]. Activation of the PDGF-R has been associated with stimulation of tumor angiogenesis in ovarian cancer and neuroblastomas (possibly by upregulation of VEGF) and recruitment and regulation of tumor fibroblasts [8]. Data from previous studies in our laboratory have shown increased levels of phosphorylated MAPK in uterine leiomyoma compared to matched myometrial tissue and in UtLM cells following treatment with IGF-I, and that IGF-I receptor signaling in leiomyoma tissue is through MAPKp44/42 [14].

Increased mRNA expression levels of collagen types I and III in the leiomyomas versus normal myometrium has been previously reported, and it was postulated that this may be due to ovarian steroids influencing the rate of ECM protein synthesis or the changing levels of locally produced growth factors or their receptors [3]. Our study showed that cocultured UtLM cells produced significantly increased levels of collagen-type I, compared to UtLM cells cultured with UtLM cells, or UtLM cells and FB cultured alone. The production of collagen type I has also been shown to be increased in cocultures of fibroblasts and MCF7 cells [36]. Furthermore, it has been shown that leiomyoma cells and normal myometrial cells treated with PDGF show upregulated expression of collagen I, as well as PCNA [34]. The increased expression of phosphorylated TGF-β receptor I and Smad2/3 in cocultured UtLM cells compared to UtLM cells cultured alone, or UtLM cells cultured with UtLM cells would suggest that activation of TGF-β/Smad pathway, in addition to increased RTK signaling, resulted in enhanced cell proliferation and increased collagen type I EMC protein and IGF-BP-3 production in cocultured UtLM cells. Others have shown an association between TGF-β and IGF-BP-3 in that airway smooth muscle cells treated with 1 ng/ml TGF-β1 had a 10-fold increase in IGF-BP-3 compared to controls [37]. Our results are also in agreement with other studies, in which the authors have found that TGF-β1 and -β3 are capable of stimulating uterine leiomyoma cell growth and production of ECM proteins [18,23,24]. Smad 3 and TGF-βR proteins have been shown to be expressed in both uterine leiomyoma and myometrium [6,38]. A study has shown that treatment with gonadotropin releasing hormone analogue alters expression of both proteins, suggesting that the TGF-β system may be important in the regulation of leiomyoma growth [6]. Microarray experiments have shown that TGF-β1 and -β3 are increased in fibroids versus myometrium, and this has been confirmed using RT-PCR [17]. TGF-β, a pleiotropic gene, is also known to play a role in fibrosis. The dysregulation of this gene in the tumors suggests that TGF-β1 and -β3 may play a key role in ECM formation/accumulation in fibroids [17].

We and others have reported that IGF-I and/or its receptor (IGF-IR) are upregulated/activated in both fibroid tumors [14,16,39] and in uterine leiomyoma cells [33,40]. Because of lack of detection of IGF-I, or activation of its receptor, in the present study, we were interested in identifying factors that may have regulated the expression and or bioavailability of this growth factor. We found that the cocultured UtLM cells produced significantly higher levels of IGF-BP-3 and IGF-BP-4, while this binding protein was expressed marginally by UtLM cells and FB cultured alone. Furthermore, IGF-BP-3 and -4 were minimally expressed by cocultured and non-cocultured normal myometrial cells, or fibroblasts cultured alone. This would suggest that UtLM are the major producers of IGF-BPs, with IGF-BP-3 being secreted more abundantly than IGF-BP-4. Our results are in agreement with another *in vitro *study in which IGF-BP-3 was found to be the most predominant binding protein in conditioned media cultures from myometrial and uterine smooth muscle cells [41]. We concluded that lack of IGF-I detection and IGF-IR activation may have been due to the high levels of IGF-BP-3 in the media, making significant levels of IGF-I ligand biologically unavailable for IGF-IR binding, or for detection by the methods used in these studies.

## Conclusions

We conclude that an *in vitro *coculturing system is a useful model to gain a better understanding of cell-cell interactions that may occur *in vivo*. We found that fibroblasts derived from uterine fibroids can stimulate uterine leiomyoma cell proliferation in culture. This growth may be mediated, in part, by autocrine and/or paracrine mechanisms that promote the synthesis of soluble factors such as GFs that are important in stimulating tumor cell proliferation and production of ECM components. Understanding the molecular mechanisms of proliferation is important in identifying cell types, signaling pathways or tumor components/compartments, such as the ECM, that can be targeted for non-surgical treatment of uterine leiomyomas.

## Methods

### Cells

#### Uterine Leiomyoma-Derived Fibroblasts (FB)

Primary cultured fibroblasts were taken from leiomyoma tissue samples obtained from women undergoing hysterectomy at Duke University Medical Center, in accordance with guidelines by the NIH Office of Human Subjects Research. The primary culture was established using a previously described enzymatic digestion method [42]. Briefly, the tissue was minced into about 1-mm cubes and placed into Dulbecco Modified Eagle's Medium/Nutrient Mixture F-12 Ham (DMEM/F12) supplemented with 10% fetal bovine serum, 1% penicillin/streptomycin and 2000 U/ml crude collagenase (Type 1A; Sigma, St. Louis, MO). Tissue digestion was for 24 h in an incubator at 37°C; however, 30 min prior to the end of incubation, 2.5% trypsin was added to the flasks. Dispersed cells were then centrifuged at 50-100 × g for 3 min. The resulting cell pellet was resuspended in medium and remained in culture for 7 days before subculturing. A FACSVantage SE flow cytometer equipped with digital electronics (Becton Dickinson) was used to sort the FB cells from smooth muscle cells with the OTR antibody (Santa Cruz Biotechnology, Inc., Santa Cruz, CA) labeling. Propdium iodide (PI) was also added to the OTR stained cells prior to examination to measure membrane integrity. The sorted cells were analyzed using BD FACSDiVa software with FB identified as OTR/PI negative. The primary cultured FB were further conformed by immunocytochemistry and immunofluorescence staining. FB were immunostained for the intermediate filament vimentin with primary antibody (BioGenex, San Ramon, CA) and a Super Sensitive link label immunohistochemistry (IHC) detection system (BioGenex) according to the manufacturer's protocol. UtSMC and UtLM cells were also stained for vimentin as positive controls. Desmin, a specific marker for muscle cells and normal mouse gamma globulin (BioGenex) served as the negative control. Furthermore, confocal (Zeiss LSM 510 meta, Carl Zeiss Inc, Oberkochen, Germany) immunofluorescence was done using a primary anti-goat OTR antibody (C-20; Santa Cruz Biotechnology) and donkey anti-goat Alexa Fluor 488 (Molecular Probes, Eugene, OR) as the secondary antibody to conform FB of not uterine smooth muscle origin according to manufacture's protocol. UtSMC and UtLM staining served as positive control, and normal goat serum served as negative control.

#### Human uterine leiomyoma (UtLM) cells

UtLM cells (GM10964) were purchased from Coriell Institute for Medical Research (Camden, NJ) and maintained in MEM (Gibco Life Technologies, Grand Island, NY) with supplements at 37°C, with 95% humidity, and 5% carbon dioxide as previously described [33].

#### Human uterine smooth muscle cells (UtSMC)

UtSMC were also purchased from Clonetics (San Diego, CA) and grown in media containing supplements and fetal bovine serum from a SmGM-2 Bullet Kit (Cambrex, Walkersville, MD) under same condition as UtLM cells.

### Cell Coculture

UtLM, UtSMC and FB cells were grown separately under their specific media conditions as described above. All cells were seeded at 5 × 10 ^4 ^cells/well onto the base of the well or onto the membrane of an overlying insert (with a 1 mm pore size) of 6-well tissue culture companion plates (BD Biosciences, Lincoln Park, NJ). The cells grew separately for approximately 2 days, and were then cocultured in the routine medium. After 24 h, the media were switched to DMEM phenol red free media with stripped serum (Hyclone, Logan, UT) for the cell proliferation assays, and DMEM phenol free and serum free for the western blot, phosphorylated receptor tyrosine kinase (pRTK) array and ELISA procedures. For the three-cell culture conditions (UtLM cells or UtSMC cultured with FB, UtLM cells or UtSMC alone, or FB alone) there were duplicate wells per condition and three independent experiments were conducted. Each well contained a total of 5 ml of medium. Since UtLM cells grow at a rate of approximately 1 population doubling every 10 days [43], the cells were allowed to incubate in coculture for 7 days. On day 7, the cells at the base of the wells were counted three times using the Coulter Counter Model Z1 (Beckman Coulter, Miami, FL) or Cellometer Auto T4 Plus Counting System (Nexecolm Bioscience LLC, Lawrence, MA). Aliquots of media samples and cell lysates from the wells were collected on day 7 in order to allow the secreted soluble proteins to accumulate to detectable levels. At 24 h, 48 h, and day 7, cell lysates were also harvested for RTK arrays or westerns of the receptors and their target proteins in order to capture their phosphorylation status after coculture. All the samples were placed in a protease inhibitor cocktail (Sigma), and frozen at -80°C until ready for use in ELISA, western blot and pRTK assays.

### Cell Proliferation

For cell proliferation assays, UtLM cells, UtSMC and FB were plated at density of 2.5 × 10 ^4 ^cells/well and allowed to attach and grow for 4 days on coverslips (Oncor, Gaithersburg, MD) placed at the bottom of each well. There were three cell culture conditions (UtLM cells or UtSMC cultured with FB, UtLM cells or UtSMC alone, or FB alone), which had duplicate wells per condition. On day 3, media were aspirated from wells, and the wells were filled with 70% ethanol for 10 min prior to a wash in 1 × automation buffer for 5 min. The cells were later fixed by placing 4% paraformaldehyde (Electron Microscopy Sciences, Hatfield, PA) in wells for 10 min, followed by a rinse with 1 × AB prior to and after incubation in 0.2% triton X-100 (Sigma) for 20 min. The coverslips containing fixed cells were then removed from the wells, and placed in a humidity chamber for staining. Incubation in a peroxidase block (BioGenex) was done for 10 min. The coverslips were washed before a 30 min incubation in a 1:75 dilution of the primary anti-mouse proliferating cell nuclear antigen (PCNA) antibody (Chemicon International, Temecula, CA) and staining was done as previously described [44]. Three independent immunocytochemical staining experiments were conducted. Percent PCNA labeling was determined by the number of cells having positively stained (brown) nuclei divided by 1,000 cells total (i.e., labeled and unlabeled) and multiplied by 100.

### ELISA

ELISA kits were used for determination of TGF-β1 (Anogen, Mississauga, Ontario, Canada), VEGF (IBL, Takasaki-shi, Gunma, Japan) and epidermal growth factor (EGF) (Antigenix America Inc., Huntington Station, NY) levels. The procedures were performed according to the manufacturers' protocols. Protein levels in the culture medium were concentrated 10 × and were measured as ng/ml or pg/ml. Four replicates of data for each growth factor were used for statistical analyses.

### Western Blot Analysis

To detect GFs in conditioned medium and their signaling proteins in cell lysates, various primary antibodies (see below) were used. The cell lysates and concentrated media (1:10 × concentration, Millipore, Billerica, MA) were used for western blotting analyses as previously described [33]. Briefly, blots were incubated overnight with a specific diluted primary antibody: 1:200 for anti-collagen-I (Calbiochem, San Diego, CA); 1:300 for anti-FGF-2 (Santa Cruz Biotechnology) and anti-IGF-BP-3 and -4 (Santa Cruz Biotechnology); 1:500 anti-PDGF-A and -B (R & D Systems, Minneapolis, MN); 1:1000 for anti-p-MAPK44/42, anti-t-MAPK44/42 (Cell Signaling Technology, Inc., Beverly, MA), anti- t-Smad-2 and p-Smad-2 (Cell Signaling Technology, Inc.), and anti- t-Smad-3 and p-Smad-3 (Cell Signaling Technology, Inc.); and 1:3000 for anti-TGF-β3 (Genex Bioscience Inc., Hayward, CA), followed by incubation with the horseradish peroxidase-conjugated secondary antibody: 1:5000 anti-mouse (Amersham Biosciences, Piscataway, NJ) for collagen-I; 1:5000 anti-rabbit (Amersham Biosciences) for FGF-2, TGF-β3, Smad-2, and MAPK44/42; or 1:7000 donkey anti-goat (Santa Cruz Biotechnology) for IGF-BP-3 and PDGF-A and -B. A densitometer (Fluor ChemTM 8900, Alpha Innotech, San Leandro, CA) was used for quantitation of band densities. Three independent experiments were conducted and three sets of data were used for statistical analysis of western blot data.

### Human phospho-Receptor Tyrosine Kinase (pRTK) Array

Expression of phosphorylated receptor tyrosine kinases (pRTKs) was detected at 48 h using a pRTK Array Kit (R&D Systems). Two hundred micrograms of total protein from lysates of cocultured UtLM cells or UtLM cultured alone were incubated with a pRTK array membrane containing anti-pRTK antibodies. The procedures were performed according to the manufacturer's protocol. Four replicates of data for each pRTK were used for statistical analyses.

### Immunoprecipitation

For the evaluation of the TGF-β/Smad pathway, immunoprecipitation was used to pull down the transforming growth factor-beta receptor I (TGF-βRI) from cell lysates (50 mg) of UtLM cells cocultured with FB, or UtLM and FB alone at 48 h using anti-TGF-βRI (1:300 dilution) (Santa Cruz Biotechnology). The precipitate was then blotted with a 1:250 dilution of anti-rabbit phosphoserine (Zymed Laboratories, Inc., San Francisco, CA). A Seize Primary Immunoprecipitation Kit (Pierce Biotechnology, Rockford, IL) was used as previously described [14].

### Statistical Analysis

Most data were not normally distributed so nonparametric statistical methods were used to compare UtLM cells or UtSMC cocultured with FB versus UtLM cells or UtSMC and/or FB in single culture. These methods included Mann-Whitney tests and median tests [45]. Mann-Whitney tests were used to determine statistically significant differences between cocultured and single cultured cells with respect to PCNA labeling, TGF-β3 and VEGF ELISA data. Median tests were used to compare single culture versus coculture cells with respect to EGF ELISA, growth factor and matrix protein intensity data. For the p- and t- MAPK 44/42 intensity data at 24 h and 48 h, which were more normally distributed, analysis of variance was used to compare measurements across UtLM cells, FB, and cocultured UtLM cell groups. To identify which groups differed, Fisher's Least Significant Differences procedure was used. For p-MAPK at day 7 and the RTK array data, the Mann-Whitney test was used to compare UtLM cells and/or FB and UtLM cells cocultured with FB.

## List of Abbreviations

DMEM/F12: Dulbecco's Modified Eagle's Medium/Nutrient Mixture F-12 Ham; ECM: extracellular matrix; EGF: epidermal growth factor; ELISA: enzyme-linked immunosorbent assay; FB: fibroblasts; FGF-2: fibroblast growth factor-2 (or fibroblast growth factor basic/FGF-b); FGF-R: fibroblast growth factor-receptor; FITC: fluorescein isothiocyanate; GAGs: glycosaminoglycans; GFs: growth factors: HPRT: hypoxanthine phosphoribosyl-transferase; IGF-I: insulin-like growth factor-I; IGF-BP-3: insulin-like growth factor-binding protein-3; IGF-BP-4: insulin-like growth factor-binding protein-4; MAPK: mitogen activated protein kinase; OTR: oxytocin receptor; p-phosphorylated; PCNA: proliferating cell nuclear antigen; PDGF: platelet-derived growth factor; PDGF-R: platelet-derived growth factor-receptor; pRTK: phosphorylated receptor tyrosine kinase; Smad: small mothers against decapentaplegic; t-: total; TGF-β: transforming growth factor-beta; TGF-βRI: transforming growth factor-beta receptor I; UtLM: uterine leiomyoma; UtSMC: human uterine smooth muscle cells; FB: uterine leiomyoma-derived fibroblasts; VEGF: vascular endothelial growth factor;

## Competing interests

The authors declare that they have no competing interests.

## Authors' contributions

ABM participated in the design of the coculture study, assisted with the coculture initial setup, assisted with counting and sorting the cells, assisted with immunofluoresence and flow cytometry procedures, analyzed and interpreted data and drafted and revised the paper. LY participated in the design of the coculture study, carried out the cell proliferation, ELISA, western blot, pRTK array and immunoprecipitation procedures, analyzed and interpreted data and drafted and revised the paper. CDS collected the tissue samples and established the primary culture of fibroblasts, assisted with counting the cells and performed the intermediate filament immunostaining. XZ helped conduct the intermediate filament staining, performed the PCNA immunostaining and also assisted with the coculture setup and cell counting. LW assisted with the coculture initial setup, assisted with the counting the cells and PCNA immunostaining. LC assisted with counting the cells, flow cytometry and immunoflouresence procedures. GEK performed the statistical analysis. DKW and SJR provided expertise in molecular endocrinology and gynecologic pathology, respectively, and assisted with initial experimental design. DD, the principal investigator, made substantial contributions to conception and experimental design of study, finalized the paper, analyzed and interpreted the data. All authors read and approved the final manuscript.

## References

[B1] Okolo S Incidence, aetiology and epidemiology of uterine fibroids Best Pract Res Clin Obstet Gynaecol 2008 22 571 588 10.1016/j.bpobgyn.2008.04.002 18534913

[B2] Marsh EE Bulun SE Steroid hormones and leiomyomas Obstet Gynecol Clin North Am 2006 33 59 67 10.1016/j.ogc.2005.12.001 16504806

[B3] Stewart EA Friedman AJ Peck K Nowak RA Relative overexpression of collagen type I and collagen type III messenger ribonucleic acids by uterine leiomyomas during the proliferative phase of the menstrual cycle J Clin Endocrinol Metab 1994 79 900 906 10.1210/jc.79.3.900 8077380

[B4] Di Lieto A De Falco M Mansueto G De Rosa G Pollio F Staibano S Preoperative administration of GnRH-a plus tibolone to premenopausal women with uterine fibroids: Evaluation of the clinical response, the immunohistochemical expression of PDGF, bFGF and VEGF and the vascular pattern Steroids 2005 70 95 102 10.1016/j.steroids.2004.10.008 15631865

[B5] Luo X Chegini N The expression and potential regulatory function of microRNAs in the pathogenesis of leiomyoma Semin Reprod Med 2008 26 500 514 10.1055/s-0028-1096130 18951332 PMC2710997

[B6] Chegini N Luo X Ding L Ripley D The expression of Smads and transforming growth factor beta receptors in leiomyoma and myometrium and the effect of gonadotropin releasing hormone analogue therapy Mol Cell Endocrinol 2003 209 9 16 10.1016/j.mce.2003.08.007 14604812

[B7] Mangrulkar RS Ono M Ishikawa M Takashima S Klagsbrun M Nowak RA Isolation and characterization of heparin-binding growth factors in human leiomyomas and normal myometrium Biol Reprod 1995 53 636 646 10.1095/biolreprod53.3.636 7578688

[B8] Ostman A PDGF receptors-mediators of autocrine tumor growth and regulators of tumor vasculature and stroma Cytokine Growth Factor Rev 2004 15 275 286 10.1016/j.cytogfr.2004.03.002 15207817

[B9] De Wever O Mareel M Role of myofibroblasts at the invasion front Biol Chem 2002 383 55 67 10.1515/BC.2002.006 11928823

[B10] Mbeunkui F Johann DJ Jr Cancer and the tumor microenvironment: A review of an essential relationship Cancer Chemother Pharmacol 2009 63 571 582 10.1007/s00280-008-0881-9 19083000 PMC2858592

[B11] Lee KH Khan-Dawood FS Dawood MY Oxytocin receptor and its messenger ribonucleic acid in human leiomyoma and myometrium Am J Obstet Gynecol 1998 179 620 627 10.1016/S0002-9378(98)70054-7 9757961

[B12] Sendemir A Sendemir E Kosmehl H Jirikowski GF Expression of sex hormone-binding globulin, oxytocin receptor, caveolin-1 and p21 in leiomyoma Gynecol Endocrinol 2008 24 105 112 10.1080/09513590701690274 17952758

[B13] Loddenkemper C Mechsner S Foss HD Dallenbach FE Anagnostopoulos I Ebert AD Stein H Use of oxytocin receptor expression in distinguishing between uterine smooth muscle tumors and endometrial stromal sarcoma Am J Surg Pathol 2003 27 1458 1462 10.1097/00000478-200311000-00009 14576480

[B14] Yu L Saile K Swartz CD He H Zheng X Kissling GE Di X Lucas S Robboy SJ Dixon D Differential expression of receptor tyrosine kinases (RTKs) and IGF-I pathway activation in human uterine leiomyomas Mol Med 2008 14 264 275 10.2119/2007-00101.Yu 18231572 PMC2215764

[B15] Flake GP Andersen J Dixon D Etiology and pathogenesis of uterine leiomyomas: A review Environ Health Perspect 2003 111 1037 1054 12826476 10.1289/ehp.5787 PMC1241553

[B16] Dixon D He H Haseman JK Immunohistochemical localization of growth factors and their receptors in uterine leiomyomas and matched myometrium Environ Health Perspect 2000 108 Suppl 5 795 802 10.2307/3454309 11035985

[B17] Leppert PC Catherino WH Segars JH A new hypothesis about the origin of uterine fibroids based on gene expression profiling with microarrays Am J Obstet Gynecol 2006 195 415 420 10.1016/j.ajog.2005.12.059 16635466 PMC4143906

[B18] Berto AG Sampaio LO Franco CR Cesar RM Jr Michelacci YM A comparative analysis of structure and spatial distribution of decorin in human leiomyoma and normal myometrium Biochim Biophys Acta 2003 1619 98 112 12495820 10.1016/s0304-4165(02)00446-4

[B19] Lefebvre MF Guillot C Crepin M Saez S Influence of tumor derived fibroblasts and 1,25-dihydroxyvitamin D3 on growth of breast cancer cell lines Breast Cancer Res Treat 1995 33 189 197 10.1007/BF00665943 7749146

[B20] Micke P Ostman A Exploring the tumour environment: Cancer-associated fibroblasts as targets in cancer therapy Expert Opin Ther Targets 2005 9 1217 1233 10.1517/14728222.9.6.1217 16300472

[B21] Hofland LJ van der Burg B van Eijck CH Sprij DM van Koetsveld PM Lamberts SW Role of tumor-derived fibroblasts in the growth of primary cultures of human breast-cancer cells: Effects of epidermal growth factor and the somatostatin analogue octreotide Int J Cancer 1995 60 93 99 10.1002/ijc.2910600114 7529213

[B22] O'Hayre M Salanga CL Handel TM Allen SJ Chemokines and cancer: Migration, intracellular and intercellular communication in the microenvironment Biochem J 2008 409 635 649 10.1042/BJ20071493 18177271

[B23] Arici A Sozen I Expression, menstrual cycle-dependent activation, and bimodal mitogenic effect of transforming growth factor-beta1 in human myometrium and leiomyoma Am J Obstet Gynecol 2003 188 76 83 10.1067/mob.2003.118 12548199

[B24] Arici A Sozen I Transforming growth factor-beta3 is expressed at high levels in leiomyoma where it stimulates fibronectin expression and cell proliferation Fertil Steril 2000 73 1006 1011 10.1016/S0015-0282(00)00418-0 10785229

[B25] Lee BS Nowak RA Human leiomyoma smooth muscle cells show increased expression of transforming growth factor-beta 3 (TGF beta 3) and altered responses to the antiproliferative effects of TGF beta J Clin Endocrinol Metab 2001 86 913 920 10.1210/jc.86.2.913 11158066

[B26] Sozen I Arici A Interactions of cytokines, growth factors, and the extracellular matrix in the cellular biology of uterine leiomyomata Fertil Steril 2002 78 1 12 10.1016/S0015-0282(02)03154-0 12095482

[B27] Wolanska M Sobolewski K Drozdzewicz M Bankowski E Extracellular matrix components in uterine leiomyoma and their alteration during the tumour growth Mol Cell Biochem 1998 189 145 152 10.1023/A:1006914301565 9879665

[B28] Wolanska M Bankowski E Fibroblast growth factors (FGF) in human myometrium and uterine leiomyomas in various stages of tumour growth Biochimie 2006 88 141 146 10.1016/j.biochi.2005.07.014 16139411

[B29] Tsai SJ Lin SJ Cheng YM Chen HM Wing LY Expression and functional analysis of pituitary tumor transforming gene-1 [corrected] in uterine leiomyomas J Clin Endocrinol Metab 2005 90 3715 3723 10.1210/jc.2004-2303 15769981

[B30] Luo X Ding L Xu J Chegini N Gene expression profiling of leiomyoma and myometrial smooth muscle cells in response to transforming growth factor-beta Endocrinology 2005 146 1097 1118 10.1210/en.2004-1377 15604209

[B31] Ding L Xu J Luo X Chegini N Gonadotropin releasing hormone and transforming growth factor beta activate mitogen-activated protein kinase/extracellularly regulated kinase and differentially regulate fibronectin, type I collagen, and plasminogen activator inhibitor-1 expression in leiomyoma and myometrial smooth muscle cells J Clin Endocrinol Metab 2004 89 5549 5557 10.1210/jc.2004-0161 15531510

[B32] Barbarisi A Petillo O Di Lieto A Melone MA Margarucci S Cannas M Peluso G 17-beta estradiol elicits an autocrine leiomyoma cell proliferation: Evidence for a stimulation of protein kinase-dependent pathway J Cell Physiol 2001 186 414 424 10.1002/1097-4652(2000)9999:999<000::AID-JCP1040>3.0.CO;2-E 11169981

[B33] Swartz CD Afshari CA Yu L Hall KE Dixon D Estrogen-induced changes in IGF-I, Myb family and MAP kinase pathway genes in human uterine leiomyoma and normal uterine smooth muscle cell lines Mol Hum Reprod 2005 11 441 450 10.1093/molehr/gah174 15879465

[B34] Liang M Wang H Zhang Y Lu S Wang Z Expression and functional analysis of platelet-derived growth factor in uterine leiomyomata Cancer Biol Ther 2006 5 28 33 16294022 10.4161/cbt.5.1.2234

[B35] Hwu YM Li SH Lee RK Tsai YH Yeh TS Lin SY Increased expression of platelet-derived growth factor C messenger ribonucleic acid in uterine leiomyomata Fertil Steril 2008 89 468 471 10.1016/j.fertnstert.2007.02.031 17482170

[B36] Noel A Munaut C Boulvain A Calberg-Bacq CM Lambert CA Nusgens B Lapiere CM Foidart JM Modulation of collagen and fibronectin synthesis in fibroblasts by normal and malignant cells J Cell Biochem 1992 48 150 161 10.1002/jcb.240480207 1618929

[B37] Cohen P Rajah R Rosenbloom J Herrick DJ IGFBP-3 mediates TGF-beta1-induced cell growth in human airway smooth muscle cells Am J Physiol Lung Cell Mol Physiol 2000 278 L545 551 10710527 10.1152/ajplung.2000.278.3.L545

[B38] Xu J Luo X Chegini N Differential expression, regulation, and induction of smads, transforming growth factor-beta signal transduction pathway in leiomyoma, and myometrial smooth muscle cells and alteration by gonadotropin-releasing hormone analog J Clin Endocrinol Metab 2003 88 1350 1361 10.1210/jc.2002-021325 12629129

[B39] Boehm KD Daimon M Gorodeski IG Sheean LA Utian WH Ilan J Expression of the insulin-like and platelet-derived growth factor genes in human uterine tissues Mol Reprod Dev 1990 27 93 101 10.1002/mrd.1080270203 1979007

[B40] van der Ven LT Gloudemans T Roholl PJ van Buul-Offers SC Bladergroen BA Welters MJ Sussenbach JS den Otter W Growth advantage of human leiomyoma cells compared to normal smooth-muscle cells due to enhanced sensitivity toward insulin-like growth factor I Int J Cancer 1994 59 427 434 10.1002/ijc.2910590323 7927953

[B41] van der Ven LT Van Buul-Offers SC Gloudemans T Bloemen RJ Roholl PJ Sussenbach JS Den Otter W Modulation of insulin-like growth factor (IGF) action by IGF-binding proteins in normal, benign, and malignant smooth muscle tissues J Clin Endocrinol Metab 1996 81 3629 3635 10.1210/jc.81.10.3629 8855813

[B42] Freshney R Culture of animal cells: A manual of basic technique 2000 New York: Wiley-Liss Incorporated

[B43] Carney SA Tahara H Swartz CD Risinger JI He H Moore AB Haseman JK Barrett JC Dixon D Immortalization of human uterine leiomyoma and myometrial cell lines after induction of telomerase activity: Molecular and phenotypic characteristics Lab Invest 2002 82 719 728 12065682 10.1097/01.lab.0000017499.51216.3e

[B44] Moore AB Castro L Yu L Zheng X Di X Sifre MI Kissling GE Newbold RR Bortner CD Dixon D Stimulatory and inhibitory effects of genistein on human uterine leiomyoma cell proliferation are influenced by the concentration Hum Reprod 2007 22 2623 2631 10.1093/humrep/dem185 17725991 PMC2366995

[B45] Conover W Practical nonparametric statistics 1999 New York: John Wiley & Sons Incorporated

